# Chitosan-TPP Nanogels for Ocular Delivery of Folic Acid: Release Profile, Corneal Permeation, and Mucoadhesion Assessment

**DOI:** 10.3390/pharmaceutics17040424

**Published:** 2025-03-27

**Authors:** Sebastián G. Bruno, Sofía M. Martínez, Camila Costa Gobbato, Daniela A. Quinteros, Agustina Alaimo, Oscar E. Pérez

**Affiliations:** 1 Departamento de Química Biológica, Facultad de Ciencias Exactas y Naturales, Universidad de Buenos Aires, Buenos Aires C1428EGA, Argentina; sbrunoprevigliano@qb.fcen.uba.ar (S.G.B.); aalaimo@qb.fcen.uba.ar (A.A.); 2 Instituto de Química Biológica de la Facultad de Ciencias Exactas y Naturales-Consejo Nacional de Investigaciones Científicas y Técnicas (IQUIBICEN-CONICET), Universidad de Buenos Aires, Buenos Aires C1428EGA, Argentina; 3 Departamento de Ciencias Farmacéuticas, Universidad Nacional de Córdoba, Córdoba 5000, Argentina; sofia.martinez@unc.edu.ar (S.M.M.); camila.costa@mi.unc.edu.ar (C.C.G.); danielaquinteros@unc.edu.ar (D.A.Q.); 4 Unidad de Investigación y Desarrollo en Tecnología Farmacéutica-Consejo Nacional de Investigaciones Científicas y Técnicas (UNITEFA-CONICET), Córdoba 5000, Argentina

**Keywords:** chitosan, folic acid, nanogels, mucoadhesion, corneal permeation

## Abstract

**Background**: Folic acid (FA) is essential for cellular functions but has limited ocular bioavailability, restricting its therapeutic effectiveness. **Objective**: To develop chitosan (CS)-based nanogels (NGs) for FA transport and release, with corneal permeation evaluation. **Methods**: NGs’ hydrodynamic diameter (Ho) and polydispersity index (PdI) were determined using dynamic light scattering (DLS). CS-FA interaction was confirmed by Fourier transform infrared (FTIR) spectroscopy, differential scanning calorimetry (DSC), and thermogravimetric analysis (TGA) was applied for the dehydrated material characterization. Scanning electron microscopy (SEM) was used to evaluate the NGs ultraestructure. In vitro drug release studies were performed using a modified Franz diffusion cell, and the release profile was fitted to obtain kinetics parameters. Mucoadhesion properties were evaluated through ζ-potential measurements. Ex vivo corneal permeation studies were conducted in rabbit corneas to compare the permeability of FA contained in NGs. **Results**: NGs presented a Ho of 312.4 ± 8.2 nm and a PdI of 0.28 ± 0.04. SEM imaging revealed spherical morphologies with minor variations in size and shape induced by FA. Lyophilized and resuspended NGs exhibited a 6.8% increase in Ho and a PdI rise to 0.42, indicating slight aggregation. In vitro drug release studies demonstrated sustained FA release, as determined by the Higuchi model. Mucoadhesion studies showed a decrease in ζ-potential from +36.9 to +18.1 mV, confirming electrostatic interactions with mucin. Ex vivo corneal permeation studies indicated that encapsulated FA permeated 2.6 times slower than free FA, suggesting sustained release. **Conclusions**: our findings demonstrate the potential of nanostructures in the form of NGs to enhance FA-loaded ocular delivery and bioavailability.

## 1. Introduction

Topical drug application to the eye is significantly hindered by the eye’s protective physiological mechanisms, particularly in the cornea, leading to substantial drug loss [1]. Natural defense mechanisms, such as rapid nasolacrimal drainage, tearing, and lacrimation, efficiently remove foreign particles, preventing most of the drug from reaching the targeted ocular tissues. Consequently, less than 5% of the administered drug penetrates the cornea and reaches intraocular tissues [2]. The cornea is composed of several layers, including the epithelium, Bowman’s membrane, stroma, Descemet’s membrane, and endothelium. The epithelium serves as the primary barrier to drug penetration, consisting of tightly packed cells interconnected by tight junctions. These junctions form paracellular pores of approximately 2 nm in diameter, significantly limiting drug diffusion from the tear film into the anterior corneal segment. The drug’s physicochemical properties, particularly its lipophilicity, play a crucial role in corneal permeability. While the epithelium and endothelium favor the passage of lipophilic molecules, the stroma is more permeable to hydrophilic substances and restricts hydrophobic compounds. Consequently, drugs with balanced lipophilicity achieve better corneal penetration. Additionally, molecular charge influences diffusion, with anionic species generally exhibiting higher permeability through the corneal layers [2,3]. To address these challenges, ocular drug delivery strategies have focused on enhancing bioavailability and prolonging the residence time of topically applied drugs in the eye [4,5].

Among the various strategies explored to enhance the residence time of drugs on the corneal surface and facilitate their permeation, mucoadhesive polymers, particularly in the form of nanoparticles (NPs), have emerged as promising options [6,7]. In this sense, chitosan (CS) is one of the most notable polymeric candidates with those properties [8,9,10]. CS is a cationic polysaccharide that exhibits several favorable biological properties, such as biodegradability, non-toxicity, biocompatibility, and strong mucosal adhesion capabilities [11]. It has been suggested that the electrostatic interaction between the positively charged amino groups of CS and the negatively charged sialic acid residues in mucus, at the physiological pH values, is the primary mechanism behind its mucoadhesion. This unique combination of characteristics makes CS a highly versatile biopolymer, ideal for use in ocular medicine [12,13].

Building on these principles, CS-based NPs, such as nanomicelles, nanosuspensions, and nanogels (NG), offer key advantages, including rapid preparation under mild conditions and efficient incorporation of bioactive compounds [10,14,15]. CS-based NG can be spontaneously synthesized through the ionic gelation method by combining CS solution with tripolyphosphate (TPP), a polyanionic cross-linker. This interaction leads to the formation of both inter- and intramolecular bonds, resulting in a stable gel network. Specifically, TPP facilitates gelation via electrostatic interactions between the positively charged NH_3_^+^ groups of CS and its negatively charged counterparts, including hydroxyl (OH^−^) and phosphate species (HP_3_O_10_^4−^ and P_3_O_10_^5−^), ensuring effective cross-linking and structural integrity [16].

Particularly, this report focuses on the encapsulation of folic acid (FA), the fully oxidized monoglutamic and synthetic form of folate (vitamin B9), which occurs naturally in vegetables, green leafy plants, citrus fruits, bananas, eggs, and liver. Also, it is commonly added to fortified foods and supplements [17]. FA (C_19_H_19_N_7_O_6_) plays a crucial role in DNA synthesis, cell division, homocysteine metabolism, and cell through its protection because of its antioxidant activities. For these reasons, FA is essential for health and the prevention of various diseases. In particular, FA deficiency contributes to elevated homocysteine levels, which are detrimental to ocular health, especially in the retina. Elevated retinal homocysteine leads to neuronal death in the ganglion cell layer, altering the retinal structure and contributing to retinal diseases such as age-related macular degeneration, glaucoma, and diabetic retinopathy [18]. Moreover, FA deficiency is associated with an increased risk of cataracts as well as chronic conjunctivitis, causing inflammation and corneal damage [19]. Overall, maintaining adequate folate levels may serve as a preventive strategy for these ocular conditions, highlighting the importance of FA in supporting eye health [20,21].

In previous reports, we carefully optimized an NG formulation using the ionic gelation (IG) method, systematically adjusting the concentrations of CS and TPP to efficiently encapsulate bioactive compounds [16,22,23]. In this context, the present study investigates the potential of FA-loaded NGs to enhance precorneal residence time, incorporating comprehensive physicochemical and biopharmaceutical characterization to assess their efficacy.

## 2. Materials and Methods

### 2.1. Materials

Medium molecular weight (MW) CS (192 kDa) [23] was from Parafarm^®^ (Saporiti S.A.C.I.F.I.A., Buenos Aires, Argentina; Ref. # 11017A). FA (99.2% purity) was generously provided by Laboratorios Bagó S.A. [24]. TPP, phosphate-buffered saline (PBS), semi-permeable cellulose acetate membrane (MWCO of 12,000 Da), and mucin from porcine stomach type III bound sialic acid 0.5–1.5% were obtained from Sigma-Aldrich Co. (St. Louis, MO, USA). Ringer’s solution (pH = 7.2) was prepared to carry out the in vitro release assays. The water employed was of Milli-Q standard (Merck Millipore, Darmstadt, Germany). All other chemicals and solvents used were of the highest purity available and were generally applied without further processing.

### 2.2. NG FA Preparation

NG FAs were prepared as previously described by Silva Nieto et al. [22] by the IG method [11]. Previously, each component stock solution was prepared. Thus, the polysaccharide was dissolved in acetic acid solution (1% *v*/*v*; pH 4.5), TPP was dissolved separately in Milli-Q water (2.5% *v*/*v*), FA (1.0 mM) was freshly prepared in PBS (pH 7.4) at the beginning of each assay.

TPP (C_f_ = 0.075% *w*/*v*; pH 9) was added dropwise to a CS solution in ultrapure water (C_f_ = 0.225% *w*/*v*; pH 4.5) under magnetic stirring at room temperature (RT). FA (C_f_ = 300 or 500 µM; pH 7.4) was added together with the cross-linker, and the mixture was stirred for 30 min. Blank NGs were prepared similarly, using Milli-Q water instead of FA. The resulting NGs were subsequently centrifuged at 13,500 rpm for 30 min, after which the supernatants were discarded, and the pellets were resuspended in the original volume adjusted according to the sample requirements for each experiment. However, for reference, Supplementary Table S1 provides a detailed overview of the volumes required for representative preparation of 10 mL. Then after, samples were high-intensity ultrasound (HIUS)-treated for 5 min at 750 W and 20 kHz frequency, with 20% amplitude. The samples in glass test tubes were placed in a glycerine-jacketed cooling bath at 0.5 °C to prevent heat buildup (Supplementary Figure S1). When it was necessary, NG suspensions were dispersed in sucrose at 3% *w*/*v* and frozen at −20 °C for 24 h. Subsequently, the samples were lyophilized for 24 h using Labconco Freezone^®^ 6 equipment (Labconco, Kansas City, MO, USA).

### 2.3. Characterization Techniques for NG FA

#### 2.3.1. NG Size and Electrokinetic Potential

The particle’s hydrodynamic diameter (Ho), polydispersity index (PdI), and zeta potential (ζ-Pot) of the lyophilized and non-lyophilized NG, were determined using photon correlation spectroscopy, also known as dynamic light scattering (DLS). For all measurements, a DLS Zetasizer^®^ ZS (Malvern Instruments S.A., Malvern, UK) equipped with a diode laser operating at a wavelength of 632.8 nm and a detection angle of 173° was used. To obtain particle size distribution and PdI, samples were contained in disposable polystyrene cuvettes (DTS0012, Malvern Instruments, Worcestershire, UK). On the other hand, to determine the ζ-potential values, samples were placed in disposable capillary cells (DTS1070, Malvern Instruments, Worcestershire, UK). For optimal readings, samples were diluted 1/10 in in deionized water at pH to keep the signal level. All measurements were performed at 25 °C (n = 3), and the obtained data were analyzed and interpreted according to previous reports [23,25].

#### 2.3.2. Scanning Electron Microscopy (SEM)

SEM analysis was conducted using a Sigma FE SEM microscope (Carl Zeiss, Oberkochen, Germany). NG and NG FA-300 samples were placed on a copper sheet, dried at RT, and shielded from light before being coated with a gold-palladium layer (70:30 *w*/*w*) using a PELCO Model 3 spray coating device [26]. Size frequency distribution was calculated from representative SEM images by employing Fiji imaging software, version v1.54f (Fiji Is Just ImageJ) [22].

#### 2.3.3. Fourier Transform Infrared (FT-IR) Spectroscopy

Infrared spectra were obtained by placing a lyophilized sample into the FT-IR spectrometer (Cary 630, Agilent Technologies, Santa Clara, CA, USA) and scanned from 4000 to 600 cm^−1^. Data were analyzed using OMNIC software (Thermo® Fisher Scientific, Waltham, MA, USA). The position and intensity of the absorption bands in the FTIR spectra were used to analyze the functional groups according to libraries and bibliographies [27].

#### 2.3.4. Thermal Properties of Dehydrated Samples: Differential Scanning Calorimetry (DSC) and Thermogravimetric Analysis (TGA)

The thermal properties of the various samples were analyzed using a DSC (TA Instruments, New Castle, DE, USA). Measurements were performed in a temperature range from 0 to 260 °C, under a dynamic atmosphere of N_2_ (50 mL/min) and with a heating rate of 10 °C/min, using non-hermetic aluminum pans. Pre-calibration was performed with indium according to the manufacturer’s recommended protocol.

For TGA, another 4 mg of each sample was placed in non-hermetic aluminum nanocapsules and subjected to an N_2_ flow of 50 mL/min, and a heating ramp of 10 °C/min in a temperature range of 25 to 250 °C. Pre-calibration of the equipment (TA Instruments, New Castle, DE, USA, Discovery model) of the temperature scale was performed with nickel according to the manufacturer’s recommended protocol. In both cases, TRIOS software (Version number 5.1.1, New Castle, DE, USA) was used for data processing [26].

### 2.4. Biopharmaceutical Evaluation

#### 2.4.1. In Vitro FA Release Studies

FA release studies were conducted using a modified Franz diffusion cell set at a temperature of 35.0 ± 0.5 °C. The diffusion cell, made of acrylic, was divided into two compartments: the donor compartment (1.0 mL) and the receptor compartment (4.0 mL) [28]. A semi-permeable cellulose acetate membrane separated the two compartments. In the donor compartment, 1 mL of NG suspension was introduced, while the receptor compartment was filled with PBS buffer continuously aerated with a 95% O_2_ and 5% CO_2_ mixture to maintain constant agitation. At predetermined intervals, 1 mL samples were extracted from the receptor compartment and replaced with the same volume with fresh PBS. The FA concentration in the receptor compartment was quantified using UV-Vis spectroscopy at λ = 282 nm, with each measurement repeated three times [25,29]. Of note, the absorbance of NG, used as excipients, was measured to avoid possible interference by the analytical method.

Different mathematical models may be used for inferring release patterns [30,31]:

In zero-order kinetics, the release is governed by the relaxation of polymer chains, while diffusion occurs at a constant rate, provided the system’s geometry remains unchanged throughout the release process:

(1) $$\frac{Mt}{M\infty }=\;k.t$$

where k is the release rate constant.

The Higuchi model describes drug release from a matrix system, where the release rate is proportional to the square root of time:

(2) $$\frac{Mt}{M\infty }=\;k.t^{1/2}$$

where $\frac{Mt}{M\infty }$ is the fraction of solute released at time and k is the release rate constant.

The Korsmeyer-Peppas model describes the drug release mechanism from polymeric systems with swelling capacity:

(3) $$\mathrm{ft}=\frac{Mt}{M\infty }={a.t}^{n}$$

where *ft* is the ratio of the absolute cumulative amount of the drug released at time *t* and at t_∞_, *a* is the constant that incorporates structural and geometric characteristics of the carrier, and n is the release exponent indicative of the drug release mechanism. Particularly, when n ≤ 0.45 indicates Fickian diffusion, 0.45–0.89 suggests non-Fickian kinetics, n = 0.89 corresponds to case II transport, and n > 0.89 signifies super case II transport.

#### 2.4.2. NG Mucoadhesion Evaluation

These assays were performed according to the protocol of Silva Nieto et al. [22]. Briefly, the mucin solution was prepared in ultrapure water to a C_f_: 2 mg/mL. Next, 1.0 mL of NG, NG FA-300, and NG FA-500 were each mixed with an equal volume of the free mucin solution.

The ζ-Pot values of mixed solution, NG + mucin (MC), were then measured at RT as described in Section 2.3.1, at the initial time (t_0h_) and after one day (t_24h_).

#### 2.4.3. Ex Vivo Trans-Corneal Permeation Studies

New Zealand white female rabbits (2–2.5 kg) were housed individually with ad libitum access to food and water and under a 12/12-h light/dark cycle. After anesthesia with phenobarbital, they were euthanized with a 10% O_2_ and 90% CO_2_ mixture. Eyeballs were enucleated for retinal evaluation, as formerly described [25,28]. All the experiments were conducted in accordance with the procedures of the Association for Research in Vision and Ophthalmology (ARVO) resolution on the use of animals in research, the European Communities Council Directive (86/609/EEC), and the Institutional Care and Use Committee of the School of Medicine Sciences, National University of Cordoba (CICUAL) (Res. CE-2021-00338577-UNC-SCT#FCM). All efforts were made to reduce the number of animals used.

Ex vivo trans-corneal permeation studies were carried out as previously described [28]. Briefly, rabbits were anesthetized with phenobarbital and euthanized using 10% O_2_ and 90% CO_2_. A modified Franz diffusion cell was employed, with the obtained cornea placed between donor and receptor compartments. The cornea’s endothelial side was in contact with PBS (4 mL), while the epithelial side faced the donor solution (1.0 mL). The chamber temperature was maintained at 35.0 ± 0.5 °C. Free FA or NG-FA samples were taken at set intervals (15–120 min). Samples were measured at FA’s maximum absorption wavelength (λ= 282 nm) by employing a Thermo®-Evolution 300 UV-Vis spectrophotometer (Thermo Scientific™, Loughborough, UK).The permeation area was 0.785 cm^2^.

A linear regression analysis of the obtained diffusion data allowed the calculation of the following parameters:

(i) Steady-state flux (J): v/A (µg/min·cm^2^), where A is the effective available tissue surface area.
(ii) Permeation rate (v): ∆Q/∆t (µg/min), where Q is the amount of FA diffuse through the cornea at time t.
(iii) Apparent Permeability Coefficient (P_app_): J/C_i_, where C_i_ is the initial drug concentration of the donor medium.

### 2.5. Statistical Analysis

Experiments were conducted in triplicate unless otherwise stated. In vitro drug release assays were analyzed using Kaleida Graph 4.0 (Williamstown, MA, USA). Results are presented as mean ± standard deviation (S.D.). When applicable, statistical analysis was performed using one- or two-way ANOVA, followed by Tukey’s post hoc test. Statistical significance was set at *p* < 0.05. The rest of the analyses were conducted using GraphPad Prism 8.3.0 (San Diego, CA, USA).

## 3. Results and Discussion

### 3.1. NG Size Distribution and PdI Determination

NG constituted by 2.25 mg/mL of CS and 0.75 mg/mL of TPP were selected for further investigation, sharing encapsulation efficiencies of 80.8 ± 1.2% for 300 µM FA (NG FA-300) and 80.6 ± 0.3% for 500 µM FA (NG FA-500).

**Figure 1:**
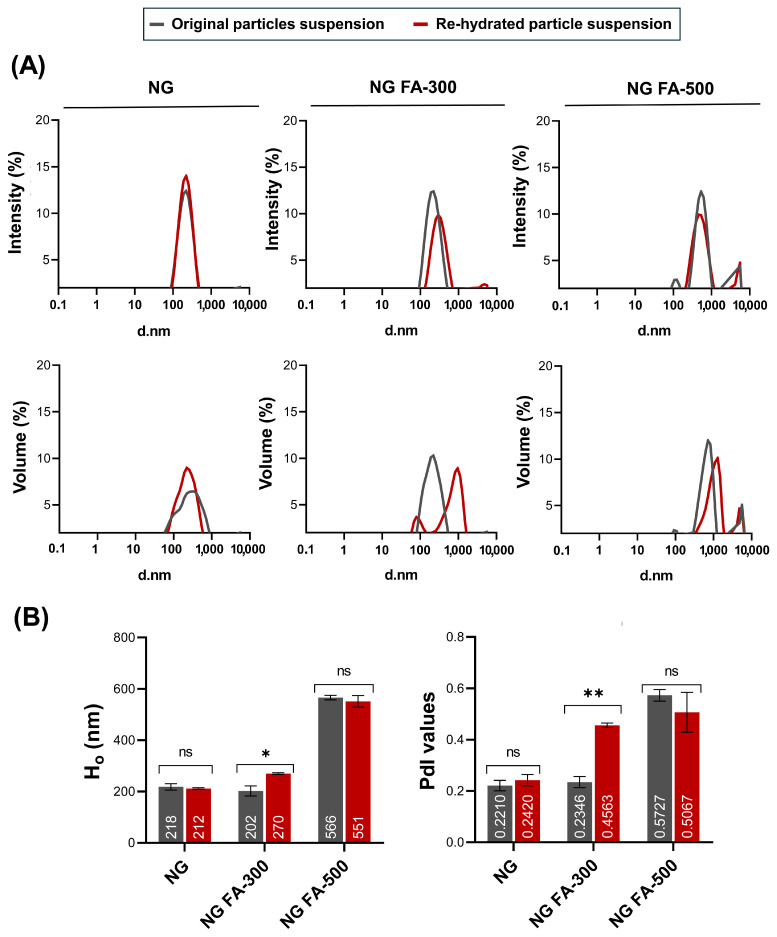
Particle size distribution **of** NG, NG FA-300, and NG FA-500 samples. (**A**) Particle size distribution profiles represented by intensity or volume. (**B**) Numbers in bars indicate the mean value reached by Ho or PdI. * *p* < 0.05 and ** *p* < 0.01; “ns” denotes non-significant differences. Grey lines/bars: original particle suspension; Red lines/bars: rehydrated particle suspension.

Ho and PdI profiles are key parameters for assessing the structural stability and functional performance of nanoformulations, especially when subjected to lyophilization and further rehydration processes. Maintaining these parameters within an optimal range ensures that the formulation retains its integrity and efficacy, which is crucial in a lyophilized drug system. Figure 1A shows that particle size distribution, whether expressed in terms of intensity or volume, remained nearly identical between the original and rehydrated NG or NG FA-500 suspensions. Also, within NG or NG FA-500, the mean values of Ho and PdI, along with their corresponding standard deviations, do not show significant variations before and after FD (Figure 1B). Notably, the NG FA-300 sample exhibited bimodal distribution profiles in both the intensity and volume graphs after rehydration (Figure 1A), which could explain the increase in PdI value. However, despite the statistically significant difference observed (*p* = 0.0211, *p* < 0.05), this variation corresponds to only a 68 nm increase in Ho (Figure 1B). Nevertheless, both Ho and PdI values remain within optimal ranges, confirming that the formulation is suitable for its use as a carrier for ophthalmic active compounds. This observation is supported by bibliographic evidence suggesting that polymodal nanosystems with particles larger than 1 µm are less effective in ocular drug delivery [32]. Optimal particle sizes are typically between 200–300 nm [33], as particles around 200 nm are efficiently absorbed topically [34]. Therefore, particle sizes near or larger than 400 nm present challenges for ocular penetration and efficacy. Additionally, the PdI quantifies the distribution of particle sizes within a sample. A low PdI signifies a narrow size distribution, while a high PdI indicates a broader range of sizes [35]. Particularly, a PdI < 0.3 is considered optimum, while values < 0.5 are within acceptable limits according to Onugwu et al. [36], who developed solid lipid NP coated with CS and poly(2-ethyl-2-oxazoline) for ocular drug delivery. These parameters should be carefully considered when selecting an optimized formulation for pharmaceutical product development [35]. Based on the present data, NG FA-300 was the top selection among the FA-encapsulated samples.

### 3.2. FTIR Analysis for Structural Characterization

**Figure 2:**
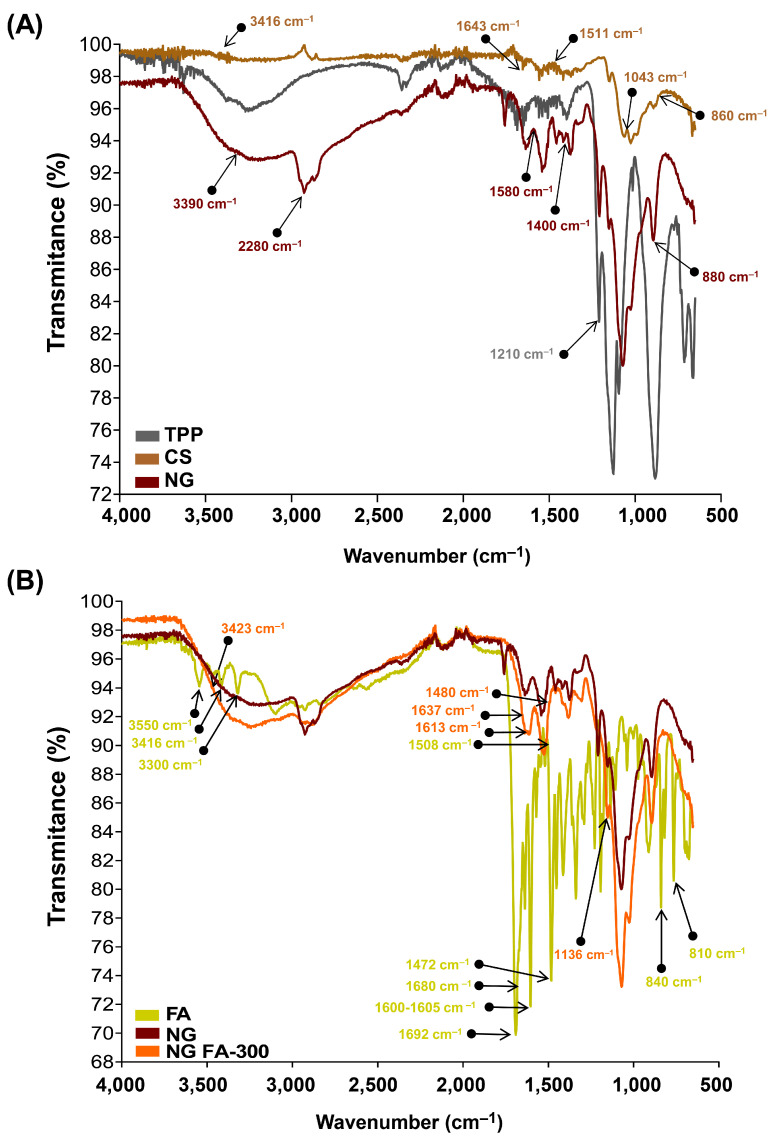
FTIR analysis in terms of transmittance (%) vs wavenumbers (cm^-1^). (**A**) TPP, CS, and NG spectra. (**B**) free FA, NG, and NG FA-300 spectra.

The chemical groups involved in the chemical interactions contributing to NG formation were investigated through the analysis of their FTIR spectra. Firstly, to facilitate the analysis, two comparisons were made: single TPP and CS versus NG (Figure 2A), and free FA versus NG FA-300 (Figure 2B).

The FTIR spectrum of TPP reveals a distinct band at 1210 cm^−1^, which is linked to the stretching vibration of P=O. Another peak at 1130 cm^−1^ corresponds to the stretching vibrations of the O−P=O group [22,37]. The spectrum of CS molecule exhibits the typical O-H stretching vibrations that appear at 3416 cm^−1^. The peaks at 1643 cm^−1^ and 1511 cm^−1^ are associated with the bending vibrations of -NH in the -NH_2_ group and C-H in the alkyl chain of the polysaccharide. The characteristic peaks for the stretching vibrations of C-O-C linkages and the glucopyranose ring in the CS structure are found at 1043 cm^−1^ and 860 cm^−1^, respectively [23]. In the NG spectrum, shifts at 3390, 2880, 1580, 1400, 1020, and 880 cm^−1^ were observed, likely caused by ionic interactions between CS and TPP [23].

The FTIR spectrum of FA exhibited several characteristic peaks (Figure 2B). De Matteo et al. [24] reported that FTIR signals for FA were sharp, more typical from a crystalline material than an amorphous one, corroborated by powder X-ray diffraction analysis. Similarly, we detected the range of 3300–3550 cm^−1^ corresponded to the stretching vibrations of -NH and -OH groups from the pterin and glutamic acid portions, respectively. A distinct stretching vibration of the carbonyl group was observed at 1692 cm^−1^, with an additional C=O stretching vibration observed at 1680 cm^−1^. The band at 1600–1605 cm^−1^ was attributed to the bending vibrations of the -NH groups, while the peak at 1472 cm^−1^, along with the absorption at 1508 cm^−1^, was linked to the C-C vibration of the pterin ring and the aromatic C=C bending of the phenyl ring, respectively. Also, a peak was detected at 840 cm^−1^ corresponding to C=C and a small vibration at 810 cm^−1^ that suggests a C-H deformation.

The FTIR spectrum of NG FA samples exhibited a significant increase in the intensity of the absorption peak at 3423 cm^−1^, attributed to the overlapping stretching vibrations of the -NH and -OH groups. Two additional bands at 1613 cm^−1^ and 1136 cm^−1^ were observed, corresponding to C-N stretching vibrations, indicating interactions of FA with CS chain, which would be primarily driven by the electrostatic forces between the cationic amino group of CS and the anionic carboxyl group of FA. Moreover, a decrease in the intensity of the FA carbonyl stretching vibration was noted, along with a slight shift in this peak. The CS’s amide band also shifted from 1643 cm^−1^ to 1637 cm^−1^, likely due to the overlapping with the newly formed C-N bond with the vitamin. The FA peak at 1508 cm^−1^, assigned as the absorption of the phenyl ring of the vitamin, was reduced in NG FA. Also, the FA shoulder next to 1508 cm^−1^ visualized in its spectrum, turns into a well-defined low-intensity peak at 1480 cm^−1^ in the NG FA spectrum. The peaks at 840 and 815 cm^−1^ associated with the FA C = C and C-H bending, respectively, are absent in the NG FA spectrum. Finally, the encapsulation of FA within the NG was confirmed by the vibrational differences between the free molecule and its entrapped form. Similarly, the NG formulation by Obrownick Okamoto-Schalch et al. [38] shows similar findings to our system.

#### SEM Analysis of Ultrastructural Features

**Figure 3:**
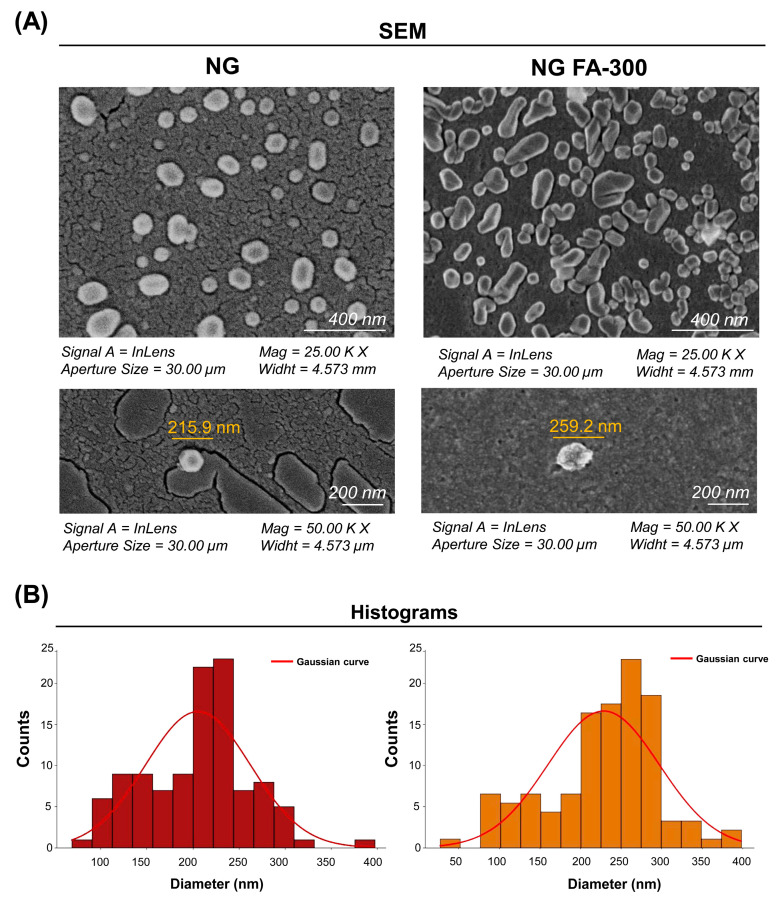
(**A**) SEM images of the NG and NG FA-300 at different magnifications, showing the NG ultrastructure and size. (**B**) Histogram representing the size distribution of both the NG and NG FA-300 nanoparticles, highlighting the average diameters. The red line corresponds to the Gaussian curve fitted to the data. Scale bar: 200 nm.

Figure 3 shows SEM images of the NG at a 200 nm scale, displaying a quasi-spherical shape, as observed by Obrownick Okamoto-Schalch et al. [38] and Silva Nieto et al. [22]. Encapsulation of FA-300 resulted in an increase in NG size, confirmed by the histogram in Figure 3B in agreement with the DLS results (Figure 1). Notably, no aggregation nor adhesion were detected in both the NG and NG FA-300. Moreover, FA encapsulation led to both spherical and rod-like shapes, with variations in size and morphology compared to control NG [36]. While Fathima et al. [39] reported spherical and smooth surfaces for NG FA, they did not mention the control NG. Therefore, it was not possible to determine whether their encapsulation process had any effect on the ultrastructure.

### 3.3. Thermal Behaviour Characterization

**Figure 4:**
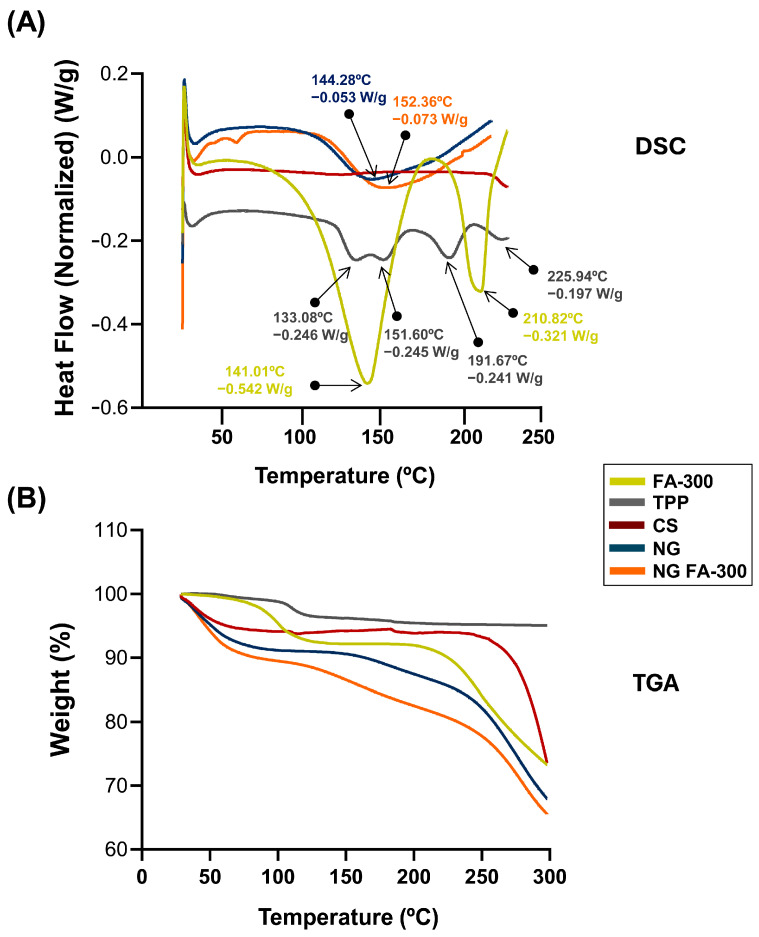
(**A**) DSC thermograms showing the heat flow profiles of FA-300, TPP, CS, NG, and NG FA-300 samples, with key thermal events, enthalpy, and transition temperature printed out by arrows. (**B**) TGA curves representing weight loss as a function of temperature, indicating thermal stability for the different analyzed samples.

DSC measures heat flow for thermal events, and TGA tracks weight changes for stability and decomposition. Both techniques analyze material properties and are common in pharmaceutics [40]. Figure 4A shows the DSC thermogram of FA in which a broad endothermic peak T_peak_1_ temperature of 141 °C indicates the melting point of the compound, while another peak T_peak_2_ appears at 210 °C confirming the loss of the amide and acid functionalities, which indicates the degradation of FA and its transformation from the crystalline to the amorphous form. This peak was determined at 177 °C by Fathima et al. [39]; meanwhile, Obrownick Okamoto-Schalch et al. [38] reported that FA was completely degraded at around 195 °C. In this sense, the degradation peak of FA in DSC can vary due to factors like heating rate, atmosphere, sample purity, and form (crystalline or amorphous). Differences in equipment calibration and environmental conditions also influence the observed temperature. These factors contribute to variations across studies [41]. Regarding CS, the characteristic peak observed at 75 °C is absent, likely due to the complete drying of the polymer sample, which removed the major part of the moisture during the dehydration process. The TPP exhibited four endothermic peaks at 133 °C, 151 °C, 191 °C, and 225 °C, which may be associated with structural changes in its crystalline form. The DSC of NG exhibited an endothermic event at 144 °C, confirming the formation of a new structure with distinct thermal properties as typical substances composed of several miscible components. When compared with NG FA-300, slight modifications in the thermal events were observed, including an endothermic peak at 152 °C, as well as the absence of the characteristic FA peaks, showing that FA is encapsulated in the NG.

Figure 4B shows the TGA analysis that indicates a loss of <8% in all samples between 28 and 100 °C. Then, the NG and NG FA-300 present a significant mass loss above 150 °C, which may be related to the endothermic peak characteristic of NG formation at 140–150 °C (Figure 4B). Comparatively, in a previous report, FA-loaded into both CS/TPP and CS/TPP/crystalline cellulose systems exhibited similar behavior attributed to water evaporation of 7.2% and 5.8%, respectively, of total weight [36].

In sum, thermal analysis showed better preservation of FA’s structure in the NG.

### 3.4. In Vitro FA Release from NG

Emerging nanotechnological methods in biopharmacy improve ocular disease treatments by enhancing targeted delivery and sustained release, reducing the need for repeated injections [42].

**Figure 5:**
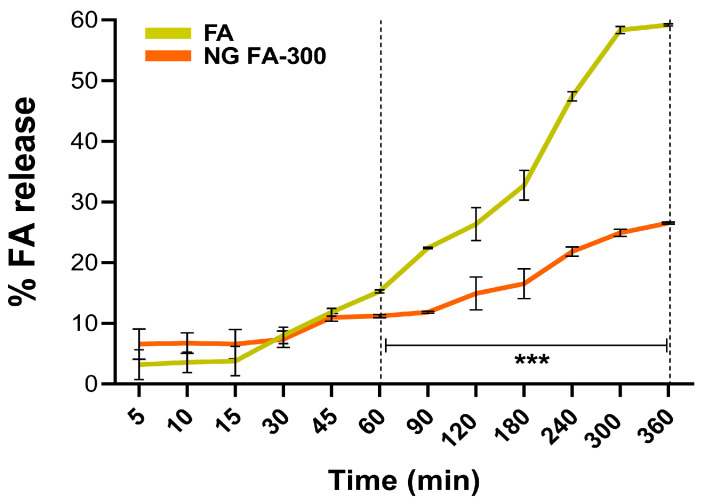
The drug release profiles of both free FA and NG FA-300 were assessed over time using a modified Franz diffusion chamber with PBS (pH 7.4) at 37 °C. Significant differences were observed from 60 min. *** *p* < 0.001.

Franz cells are commonly used to assess the release kinetics of pharmaceutical systems, especially those for controlled release of bioactive molecules [43]. Figure 5 shows that the slope of the curve (% of release versus time) of the free FA increases more sharply over time compared to its encapsulated form in NGs. In particular, the percentage of FA release differed significantly between free FA and FA in NG form, from 60 min until the end of the measurement at 360 min. This means that the FA release process occurs in a slower and more sustained manner over time compared to the free form. The most comparable study on FA release from a nanosystem in a Franz cell is by Kapoor et al. [44], who used liposomes for transdermal FA delivery to treat micronutrient deficiencies.

To estimate FA release from the NG, various mathematical models were applied to characterize the release kinetics and transport mechanisms [45]. The correlation coefficient R^2^ values, derived from applying in vitro release data to kinetic models (Table 1), range from 0 to 1, with 1 indicating a perfect fit and 0 signifying no predictive power [46]. Mathematical analysis determined that FA release from the NG follows the Higuchi model, as indicated by an R^2^ value of 0.951. This suggests that 95% of the variance in drug release is explained by this model, highlighting that the release of FA from NG is primarily governed by a diffusion-controlled mechanism. Specifically, the Higuchi model assumes that drug release occurs primarily via Fickian diffusion, whereby drug molecules migrate from regions of high concentration to lower concentration within the matrix. The high R^2^ value (0.951) further reinforces the strong predictive capacity of this model, indicating its reliability in forecasting drug release under similar experimental conditions. Notably, the Korsmeyer–Peppas model cannot be ruled out since R^2^ was 0.917. This model indicates that the release mechanism of FA was predominantly controlled by diffusion. Also, since n coefficient was ≤0.5 (0.441), a FA release was driven by the concentration gradient, according to Fickian diffusion [31].

### 3.5. Mucoadhesive Properties of NG FA

Mucoadhesive materials exhibit the ability to adhere to mucosal surfaces in areas like the gastrointestinal, reproductive, tracheobronchial, and ocular regions [47]. CS’s mucoadhesive properties are well-known, relying on hydrogen bonding and electrostatic interactions between CS and mucin (MC), as well as its molecular structure and hydrophobic nature [12,48].

**Figure 6:**
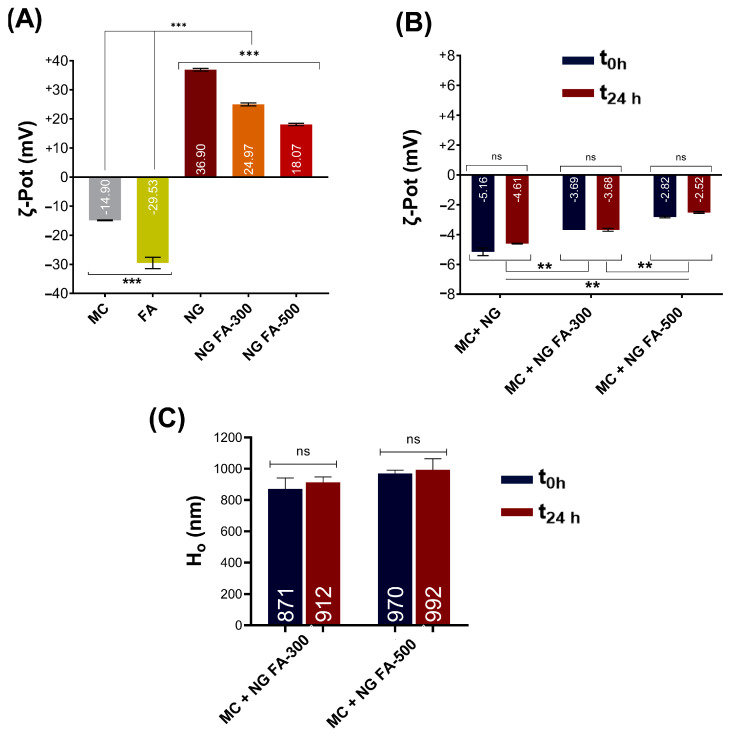
(**A**) ζ-Pot of MC, FA, NG, NG FA-300, and NG FA-500. (**B**) ζ-Pot values used as an indicator of mucoadhesion between MC and NGs over 24 h. (**C**) Ho values of MC-NGs mixed system over 24 h. Significant differences are indicated: ** *p* < 0.01, *** *p* < 0.001, ns = not significant. Blue bars represent the ζ-Pot measured immediately after mixed system preparation, while red bars correspond to measurements taken 24 h later. The values within the bars correspond to mean values.

In the present report, the ζ-Pot of MC, FA, NG, and NG FA were determined (Figure 6A). FA reduced the ζ-Pot of NGs from +36.9 mV to +24.9 mV (FA-300) and +18.1 mV (FA-500), similar to Agrawal et al. [49]. The ζ-pot profiles of each sample were presented in Figure S1, providing a detailed analysis of the surface charge characteristics. Similar results were described by de Britto et al. [50] for vitamin B9 or B12-loaded NG. Also, FA-encapsulated CS-TPP/cellulose nanocrystals showed +25 mV [26]. Subsequently, the NG FA samples were incubated with the MC solution. The ζ-Pot values of the NG-MC mixed systems indicate the presence of interactions between the biopolymer and the heavily glycosylated protein (Figure 6B). The detected formation of NG-MC was confirmed by the observed increase in hydrodynamic size (Ho) compared to each sample analyzed individually (Figure 1A versus Figure 6B,C).

It was found that the electrophoretic potential of the NGs decreased when in contact with MC, lasting at least 24 h. As FA concentration increased, the ζ-Pot of the NG-MC system approached zero, with the initial ζ-Pot being lower in the presence of FA.

The mucoadhesive capacity of NGs helps to prolong residence time and sustain the release of the active ingredient [51]. Therefore, it can be concluded that our generated NG exhibits these biopharmaceutical properties. Notably, this would be the first report providing strong evidence of the mucoadhesive properties of NGs for FA as a therapeutic agent. These findings would be an advance in drug delivery, especially for therapies needing prolonged retention on mucosal surfaces like ocular tissue.

### 3.6. Ex Vivo Assays: Trans-Corneal Permeation Studies

**Figure 7:**
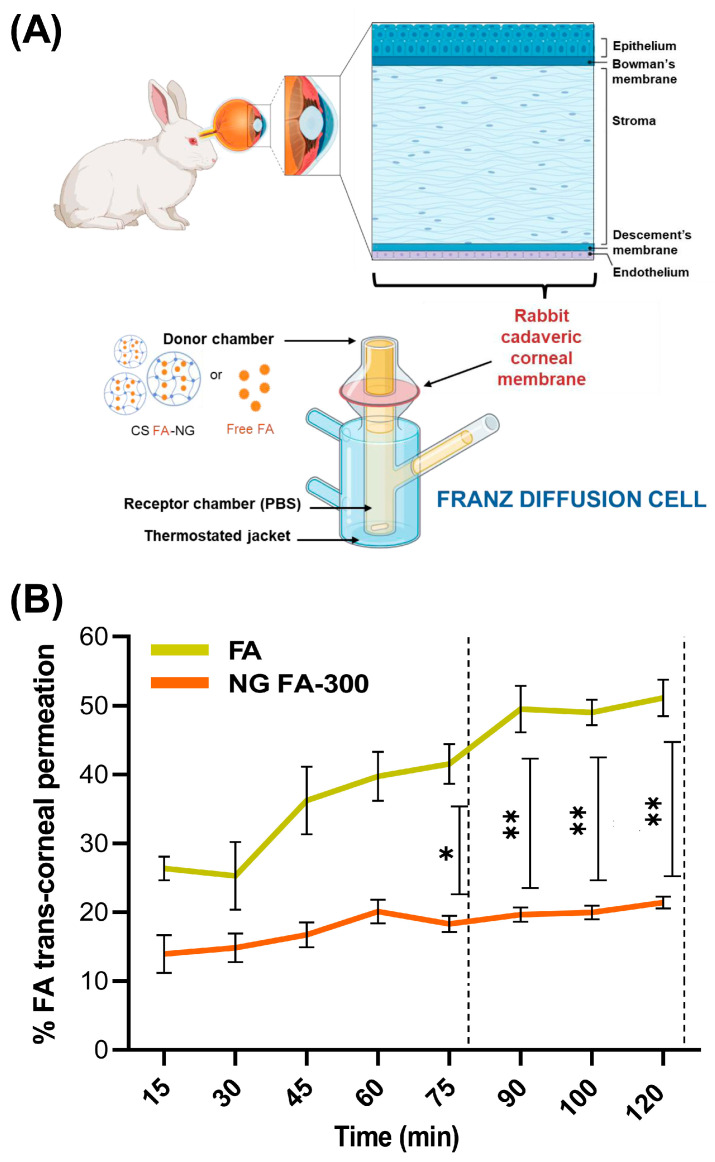
(**A**) Illustration of the trans-corneal permeation setup in which rabbit cadaveric corneal membranes and a modified Franz diffusion chamber were employed. The experimental setup allows for testing the permeation of both free FA and NG FA-300. Illustration created using the BioRender platform. (**B**) Trans-corneal permeability parameters for both free FA and NG FA-300 over time. * *p* < 0.05 and ** *p* < 0.01.

Figure 7A resents permeation assays for both free FA and NG FA-300, conducted using an adapted Franz bicompartmental chamber with corneal tissue extracted from *New Zealand* white female rabbits.

Figure 7B presents the results of the trans-corneal and permeation tests; meanwhile, Table 2 displays flux (J) and apparent permeability coefficient (P_app_) parameters. Data obtained demonstrated that NG FA permeated through the cornea at a rate 2.6 times slower than the free FA solution. Notably, the higher permeation percentage of free FA compared to the form into NG exhibited a remarkable and statistically significant difference from 75 min until the conclusion of the measurement at 120 min. Similarly, Martínez et al. [25] reported the use of human serum albumin as a nanocarrier for melatonin delivery. Due to its low solubility, melatonin faces challenges in permeation. Using a Franz chamber and ex vivo corneal tissues, the authors found that melatonin-loaded albumin NP showed lower permeation compared to the free neurohormone. Kapoor et al. [44] studied the skin penetration of FA-loaded liposomes for cosmetic applications using a Franz diffusion chamber. The used nanocarriers showed 8 times higher penetration than the FA solution, with the difference attributed to the cornea’s selective barrier, which limits diffusion compared to the skin [2,52,53].

To sum up, FA encapsulated in NG showed a lower but more sustained permeation rate than the free vitamin. This formulation would serve as an effective reservoir, enabling gradual and prolonged drug release, which could help maintain therapeutic FA levels, enhance efficacy, and reduce dosing frequency. Furthermore, this approach not only mitigates the initial burst release commonly observed in conventional drug solutions but also enhances drug stability and bioavailability [54].

## 4. Conclusions

The present report highlights the potential of NG FA as a promising ophthalmic drug delivery system. The NG FA formulation maintained its optimal size post-lyophilization and rehydration, with only minimal variations that remained. Through FTIR analysis, interactions at a level of chemical groups between FA and the CS were confirmed, while DSC and TGA analysis showed the thermal properties of dehydrated NGs. In vitro release studies revealed that NG FA provided a slower and more sustained release profile compared to the free vitamin, which could result in prolonged therapeutic effects, reducing the need for frequent administrations. Furthermore, the mucoadhesive properties of NG FA indicated their ability to adhere to ocular surfaces, leading to extended FA retention and enhanced bioavailability at the target site. This prolonged contact time could contribute to improved treatment outcomes by ensuring steady FA permeation over time. Collectively, these findings underscore the potential of FA-loaded NG as a controlled-release system that would enhance therapeutic efficacy, optimize ocular drug delivery, and support the development of innovative treatments for ophthalmic diseases.

## Figures and Tables

**Table 1:** Parameters obtained from drug release profile fitting for both free FA and NG FA.

System	Mathematical Models of Drug Release						
	Zero-Order Plot		Higuchi Model		Korsmeyer–Peppas Model		
	*k* (min)	R^2^	*k* (min^−1/2^)	R^2^	*k* (min^−n^)	n	R^2^
**Free FA-300**	12.681 ± 0.520	0.978	20.072 ± 1.290	0.947	15.938 ± 0.228	0.781 ± 0.013	0.999
**NG FA-300**	4.996 ± 0.523	0.483	8.263 ± 0.294	0.951	8.651 ± 0.472	0.441 ± 0.056	0.917

Data shown as mean ± S.D.; n = 3.

**Table 2:** Comparison of permeation properties between free and encapsulated FA.

System	Steady-state Flux (J) (μg/min)	Permeated (μg) (After 2h)	Apparent Permeability Coefficient, P_app_ (cm/min) (×10^−4^)
**free FA-300**	0.113 ± 0.004	33.848 ± 2.187	2.123 ± 0.032
**NG FA-300**	0.0427 ± 0.003	14.611 ± 0.627	0.824 ± 0.055

Data shown as mean ± S.D.; n = 3.

## Data Availability

Data will be made available upon request.

## Supplementary Materials

The following supporting information can be downloaded at: https://www.mdpi.com/article/10.3390/pharmaceutics17040424/s1, Figure S1: Basic scheme of FA-loaded NG generation. Control: NG without FA. Illustration created using BioRender platform; Figure S2: ζ-Pot profiles of NG, NG FA-300, NG FA-500, and MC, along with those of the complexes formed between MC and each NG sample. For the complexes, ζ-Pot measurements were taken at t0h and t24h to evaluate surface charge variations over time; Table S1: Volumes used for the generation of NG.

## Abbreviations

The following abbreviations are used in this manuscript:

CS	chitosan
DD	degree of deacetylation
DLS	dynamic light scattering
DSC	differential scanning calorimetry
FA	folic acid
FT-IR	Fourier transform-infrared spectroscopy
HIUS	high-intensity ultrasound
IG	ionic gelation
Ho	hydrodynamic diameter
MC	mucin
MW	molecular weight
NG	CS-based nanogels
NG FA-300	folic acid (300 µM)-loaded chitosan-based nanogels
NG FA-500	folic acid (500 µM)-loaded chitosan-based nanogels
NP	nanoparticles
PdI	polydispersity index
RT	room temperature
SEM	scanning electron microscopy
TGA	thermogravimetric analysis
TPP	sodium tripolyphosphate
ζ-Pot	ζ-potential
