# Supplementary material for: Chitosan-TPP Nanogels for Ocular Delivery of Folic Acid: Release Profile, Corneal Permeation, and Mucoadhesion Assessment
Source: Pharmaceutics. 2025 Mar 27;17(4):424. doi: 10.3390/pharmaceutics17040424 (PMC12030068; doi:10.3390/pharmaceutics17040424)
Supplement: Supplementary file 1 [file pharmaceutics-17-00424-s001.zip › New_Supplementary Table 1.docx]

**Supplementary Table 1.** Volumes used for the generation of NG

| **System** | **CS (mL)** | **TPP (mL)** | **FA (mL)** | **Milli Q- H_2_O (mL)** |
| --- | --- | --- | --- | --- |
| **NG** | 2.25 | 0.3 | 0 | 7.45 |
| **NG FA-300** | 2.25 | 0.3 | 3 | 4.45 |
| **NG FA-500** | 2.25 | 0.3 | 5 | 2.45 |

*Volumes expressed for the preparation of a final 10 ml for each system*
