# Supplementary figures and images for: Chitosan-TPP Nanogels for Ocular Delivery of Folic Acid: Release Profile, Corneal Permeation, and Mucoadhesion Assessment
Source: Pharmaceutics. 2025 Mar 27;17(4):424. doi: 10.3390/pharmaceutics17040424 (PMC12030068; doi:10.3390/pharmaceutics17040424)

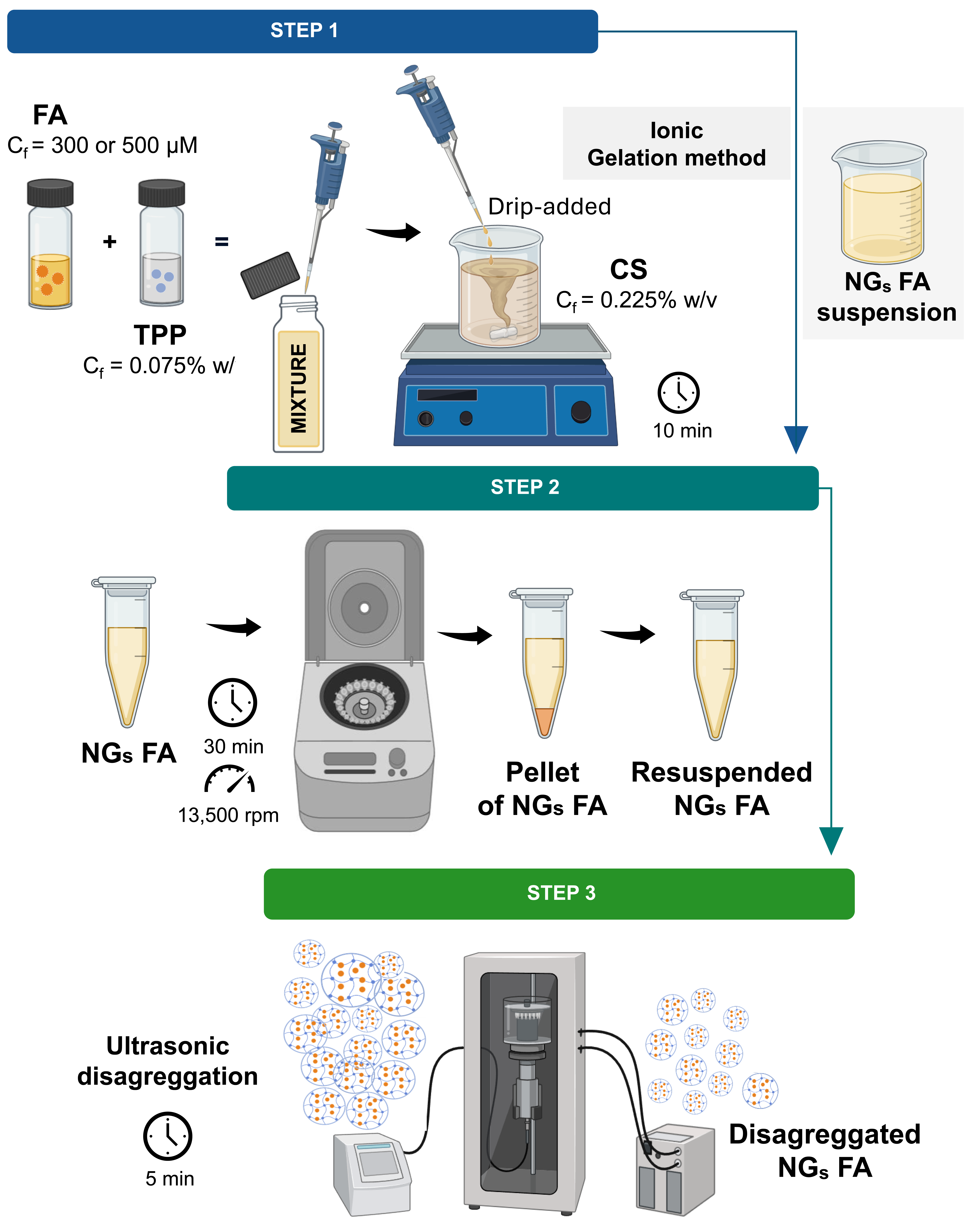

Supplement: Supplementary file 1 [file pharmaceutics-17-00424-s001.zip › Figure 1_Supplementary.tif]

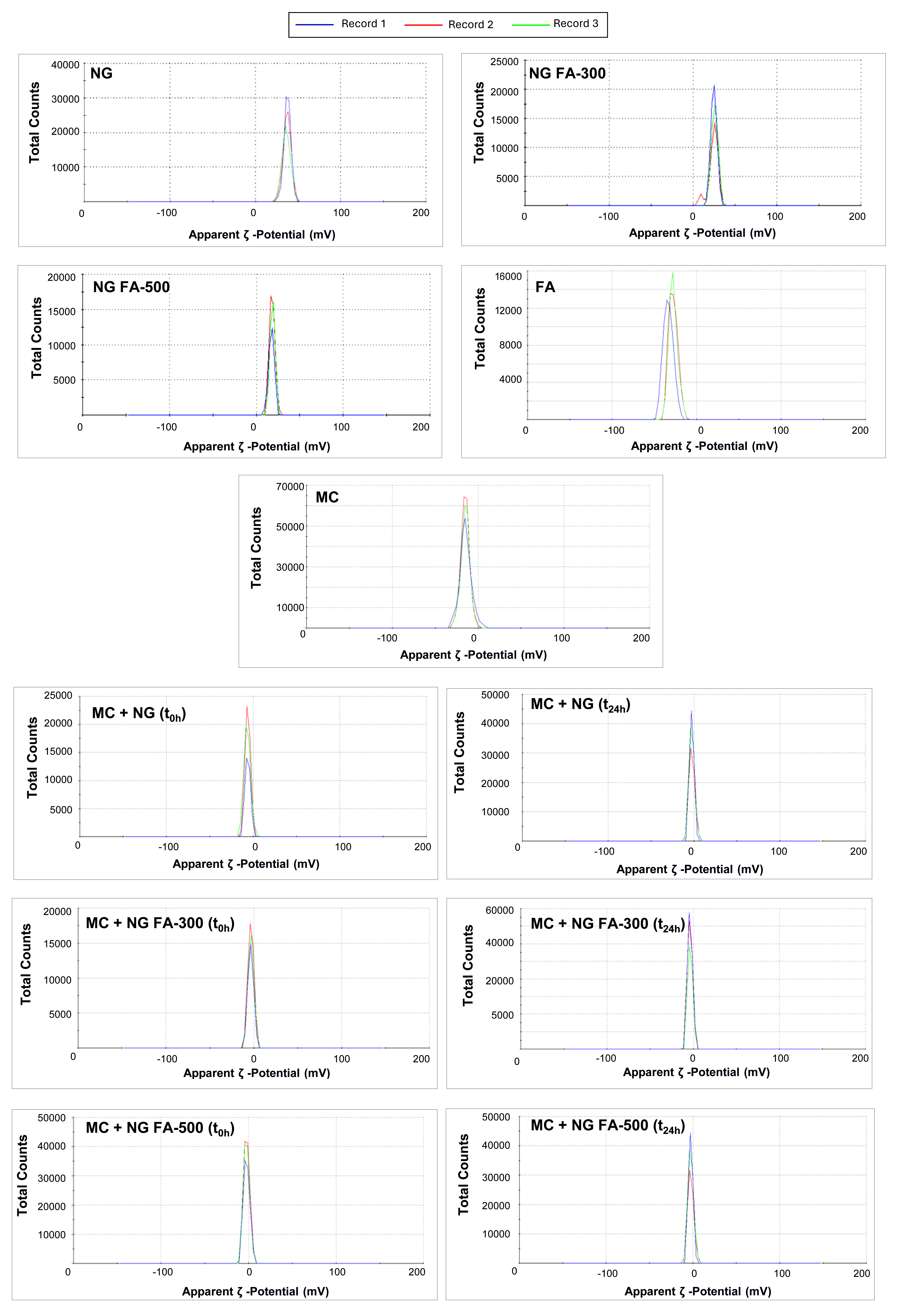

Supplement: Supplementary file 1 [file pharmaceutics-17-00424-s001.zip › Figure 2_Supplementary.tif]
